# Integrated miRNA-mRNA Expression Profiles Revealing Key Molecules in Ovarian Cancer Based on Bioinformatics Analysis

**DOI:** 10.1155/2021/6673655

**Published:** 2021-10-25

**Authors:** Chao Li, Zhantong Hong, Miaoling Ou, Xiaodan Zhu, Linghua Zhang, Xingkun Yang

**Affiliations:** Department of Obstetrics Laboratory, Foshan Women and Children Hospital Affiliated to Southern Medical University, Foshan, Guangdong 528000, China

## Abstract

Ovarian cancer is one of the leading causes of gynecological malignancy-related deaths. The underlying molecular development mechanism has however not been elucidated. In this study, we used bioinformatics to reveal critical molecular and biological processes associated with ovarian cancer. The microarray datasets of miRNA and mRNA expression profiles were downloaded from the Gene Expression Omnibus (GEO) database. Besides, we performed target prediction of the identified differentially expressed miRNAs. The overlapped differentially expressed genes (DEGs) were obtained combined with miRNA targets predicted and the DEGs identified from the mRNA dataset. The Cytoscape software was used to design a regulatory network of miRNA-gene. Moreover, the overlapped DEGs in the network were subjected to enrichment analysis to explore the associated biological processes. The molecular protein-protein interaction (PPI) network was used to identify the key genes among the DEGs of prognostic value for ovarian cancer, and the genes were evaluated via Kaplan-Meier curve analysis. A total of 186 overlapped DEGs were identified. Through miRNA-gene network analysis, we found that miR-195-5p, miR-424-5p, and miR-497-5p highly exhibited targeted association with overlapped DEGs. The three miRNAs are critical in the regulatory network and act as tumor suppressors. The overlapped DEGs were mainly associated with protein metabolism, histogenesis, and development of the reproductive system and ocular tissues. The PPI network identified 10 vital genes that promote tumor progression. Survival analysis found that CEP55 and CCNE1 may be associated with the prognosis of ovarian cancer. These findings provide insights to understand the pathogenesis of ovarian cancer and suggest new candidate biomarkers for early screening of ovarian cancer.

## 1. Introduction

Ovarian cancer is one of the leading causes of deaths resulting from gynecological malignancies. The latest statistics indicate that about 295,414 new cases of ovarian cancer were reported globally in 2018 [1]. The overall 5-year survival rate of ovarian cancer is below 45% mainly because distant metastasis occurs earlier before diagnosis. Biomarkers such as CA125 are currently in clinical use; however, they are unspecific, and ultrasound examinations cannot identify early cases [2, 3]. Therefore, there is an urgent need to reexcavate new diagnosed biomarkers for ovarian cancer and reidentify the associated key molecules. This will be vital in devising strategies to manage ovarian cancer at prevention and control levels.

In recent years, the extensive application of expression profiles has accumulated enormous omics data, which is dependent on in-depth interpretation. Following relevant researches in the past three years, a few reports on the use of expression profiles linked with bioinformatics to discover key genes of ovarian cancer have been published [4–7]. However, most of the research groups selected similar microarray profiles thereby may cause lower accuracy and gave false-positive results. The miRNAs are noncoding RNAs that bound to complementary sequences in the mRNA via base pairing. This promotes mRNA silencing and negatively regulates downstream gene expression [8]. Many studies have found that miRNA disorders can occur in nearly all types of tumors thereby affecting target gene expression [9, 10]. A number of studies have evaluated that miRNA expression profiles in patients with ovarian cancer can be used as molecular markers of malignant tumors. Functional experiments related to ovarian cancer have confirmed that many miRNAs have cancer-promoting or antitumor effects, and miRNAs can inhibit the translation process of target mRNAs to participate in the regulation of many cell processes related to ovarian cancer [11–13].

Therefore, our study adopted integrated miRNA and mRNA microarray expression profiles for joint analysis. Through bioinformatics, we constructed regulatory networks to identify key molecules and biological processes associated with ovarian cancer. This provides a scientific and accurate theoretical basis to elucidate the mechanism of ovarian cancer onset.

## 2. Materials and Methods

### 2.1. Data Sources

We searched for the microarray expression profiles of ovarian cancer in the GEO (a public functional genomics database) by limiting the sample size to more than 10 and compared tumor tissue with normal tissue for the experiment type. GSE83693 and GSE36668 were the two eligible profile datasets. The former was a miRNA profile that included 4 normal tissues and 16 tumor tissues whereas the latter was an mRNA profile that included 4 normal tissues and 8 tumor tissues.

### 2.2. Data Processing

#### 2.2.1. Differential Expression

For the mRNA dataset, the probe ID was converted to the corresponding gene name following its platform annotation file. The “limma” package of R language was used to analyze the DEGs [14], where adj.p.val < 0.05 and the absolute value of log_2_FC > 2 were defined as a statistically significant expression. Further, we used the R package “http://org.Hs.eg.db/” to convert the gene name to the corresponding gene ID [15] and eventually performed the subsequent enrichment analysis. The miRNA dataset was processed using the same method and standard.

#### 2.2.2. miRNA Target Prediction

The Funrich software 3.1.3 was used to predict the downstream targets of the identified differentially expressed miRNAs [16]. The predicted gene list was intersected with the DEGs identified from the mRNA dataset. Thus, the overlapped DEGs were generated for subsequent regulatory networks, used to identify the key genes, and for functional enrichment analysis and so on.

#### 2.2.3. Regulatory Network

The miRNAs negatively regulate target genes; therefore, the combinations of matching miRNA-gene were screened to construct regulatory networks and were visualized using the Cytoscape 3.7.1 software.

#### 2.2.4. Key Genes

The string database was used to predict the interaction network between proteins encoded by the DEGs [17]. Then, the cytohubba module in the Cytoscape software was used to identify key genes [18]; here, the source data obtained from the network file was generated via the string database. The MCC algorithm of the module was selected to identify the top 10 key genes.

#### 2.2.5. Functional Enrichment Analysis

The R package “clusterProfiler” was used to perform the GO and KEGG enrichment annotation of the overlapped DEGs [15]. GO annotation was grouped into three subcategories: molecular function (MF), biological process (BP), and cellular components (CC). KEGG is a comprehensive database that integrates genomic, chemical knowledge, and system functional information, which we used for enrichment annotation of gene pathways. The *p* value cutoff = 0.05 of the R package's parameter was considered statistically significant.

#### 2.2.6. Subsistence Analysis

The TCGA database aided in the diagnosis, treatment, and prevention of tumors through a shared mechanism. The cbioportal is a visual analytics platform developed based on the TCGA. To further evaluate the pathogenesis of key genes we obtained, the cbioportal was adopted to identify the association of key genes with the survival prognosis of ovarian cancer [19].

#### 2.2.7. Expression Level Verification

GEPIA2 is an updated version of GEPIA for analyzing the RNA sequencing expression data of 9,736 tumors and 8,587 normal samples from the TCGA and the GTEx projects, using a standard processing pipeline [20]. GEPIA2 provides customizable functions such as tumor/normal differential expression analysis, profiling according to cancer types or pathological stage, similar gene detection, correlation analysis, and dimensionality reduction analysis. Through this database, we verified whether the expression levels of candidate genes in our study were consistent.

## 3. Results

### 3.1. Differential Expression

**Figure 1:**
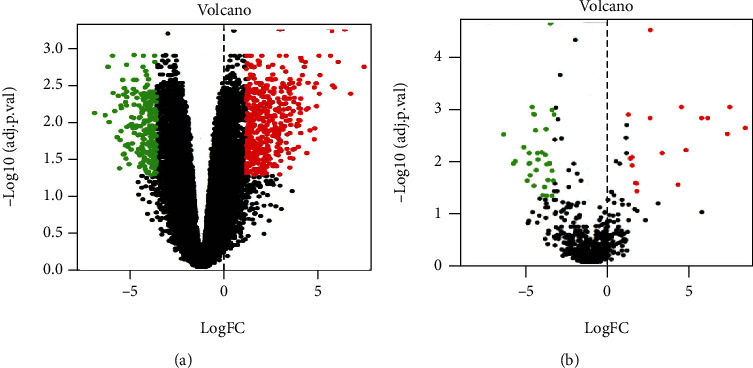
Volcanic map distribution of differential expression: (a) differentially expressed miRNA map; (b) differentially expressed gene map. Horizontal axis: log_2_(FC); vertical axis: -log_10_(adj.P.Val). The FC represents the fold change in expression of tumor samples compared to normal samples, and the adj.P.Val represents the calibrated *p* value. Green color indicates that differential expression is downregulated; red color indicates that differential expression is upregulated.

A total of 53 differentially expressed miRNAs were identified using the GSE83693 dataset, out of which 35 were downregulated, while 18 were upregulated. Besides, 680 differentially expressed mRNAs were identified using the GSE36668 dataset, out of which 239 and 441 mRNAs were downregulated and upregulated, respectively. The differentially expressed molecules are shown in Figure 1.

### 3.2. Overlapped DEGs and Regulatory Networks

**Figure 2:**
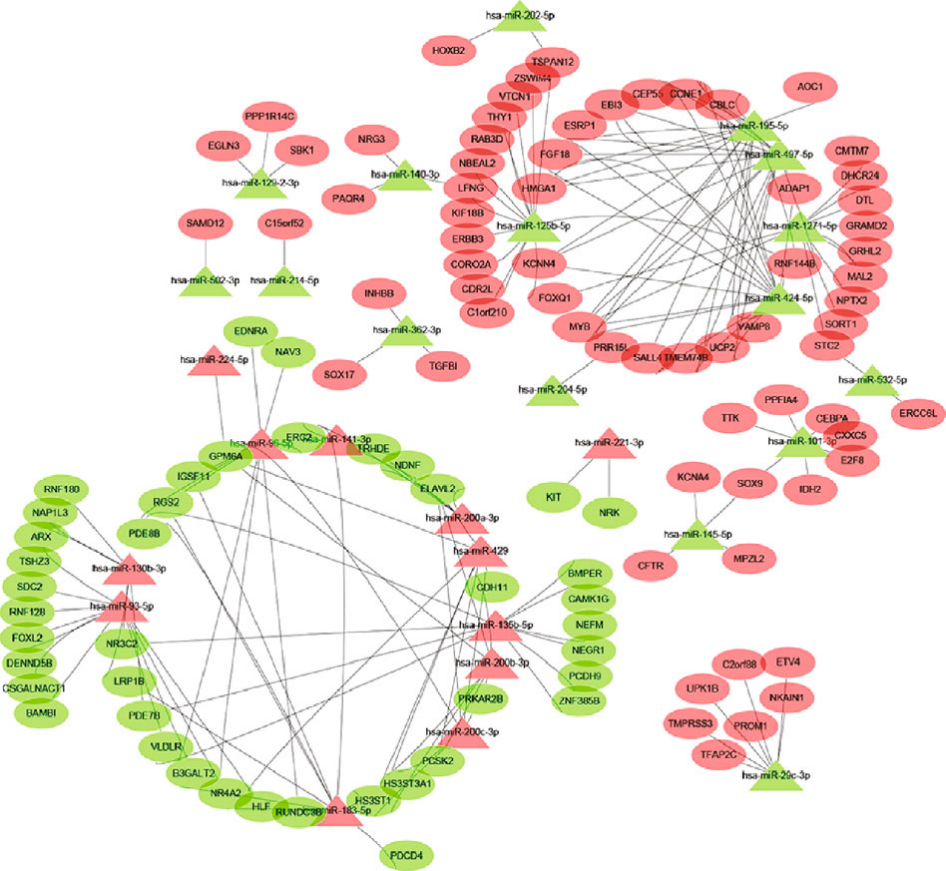
The regulation network of miRNA-gene. The ovals represent differential target genes; the triangles represent differential miRNAs; the lines represent the existence of targeted regulatory relationships. The red color shows upregulation while the green color shows downregulation.

According to the DEGs of mRNA dataset, combined with the target genes of differentially expressed miRNA predicted by the Funrich software, the 186 overlapped DEGs' network files were obtained (attachment S1 and S2 shown). The network files were imported into the Cytoscape software for visual analysis (Figure 2). It was found that miR-195-5p, miR-424-5p, and miR-497-5p were in the hub core of network regulation, of which number of target genes were the most. All three key miRNAs had 16 target DEGs.

### 3.3. Identifying the Key Genes

**Figure 3:**
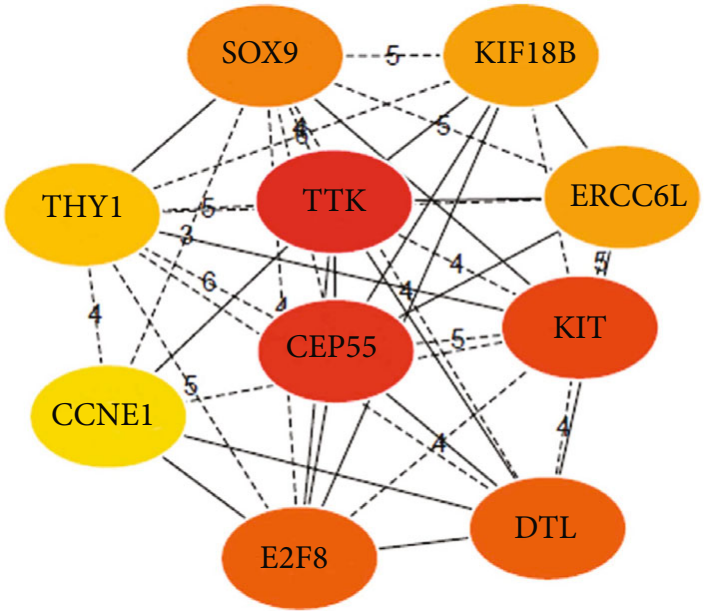
Identifying the key DEGs. When the red color appears darker, a higher score is noted, indicating a highly significant biometric significance.

The results showing the identified key genes in the overlapped DEGs via the string database and cytohubba module are shown in Table 1. All the key genes were overexpressed differential genes, of which expressions were consistent with the GEPIA2 database. The top 10 key genes were screened following the latest MCC algorithm, namely, TTK, CEP55, KIT, DTL, E2F8, SOX9, ERCC6L, KIF18B, THY1, and CCNE1. Notably, TTK, CEP55, and KIT showed the highest scores and were at the key core of the network (Figure 3).

### 3.4. Functional Enrichment Annotation

**Figure 4:**
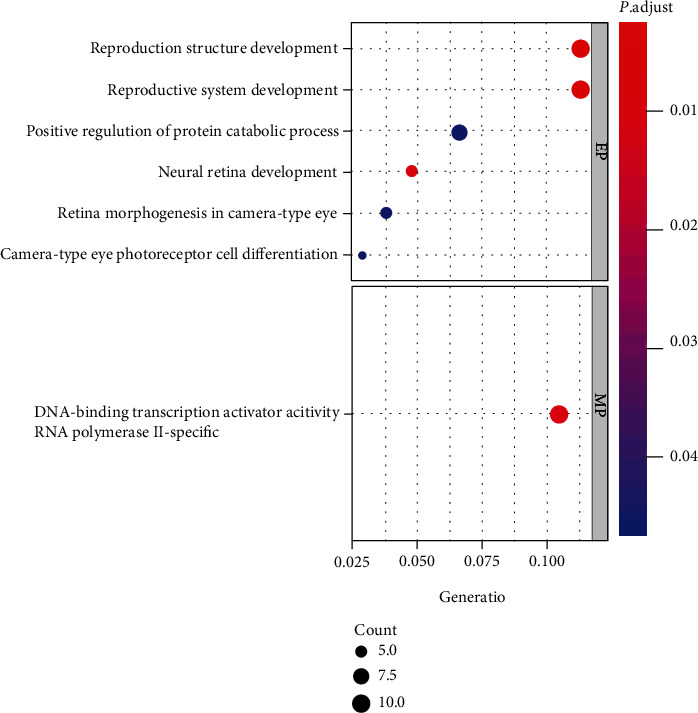
Bubble chart of GO enrichment. Horizontal axis: the proportion of genes; vertical axis: enrichment items. The color of the point corresponds to the value of *p* adjust, while the size of the point corresponds to the number of DGEs under the GO entry.

The results of GO enrichment analysis using the “clusterProfiler” package are shown in Figure 4. In BP, the DEGs were significantly enriched in developing the reproductive structure, remodeling of the reproductive system, positive regulation of protein catabolism, retinal morphogenesis, and differentiation pathways of the ocular photoreceptor cell. In MF, DEGs were enriched in DNA-binding transcriptional activation. Of note, since we set the filtering parameter at (*p* value cutoff = 0.05), KEGG analysis did not enrich the entries with coherent biological meaning.

### 3.5. Survival Analysis

**Figure 5:**
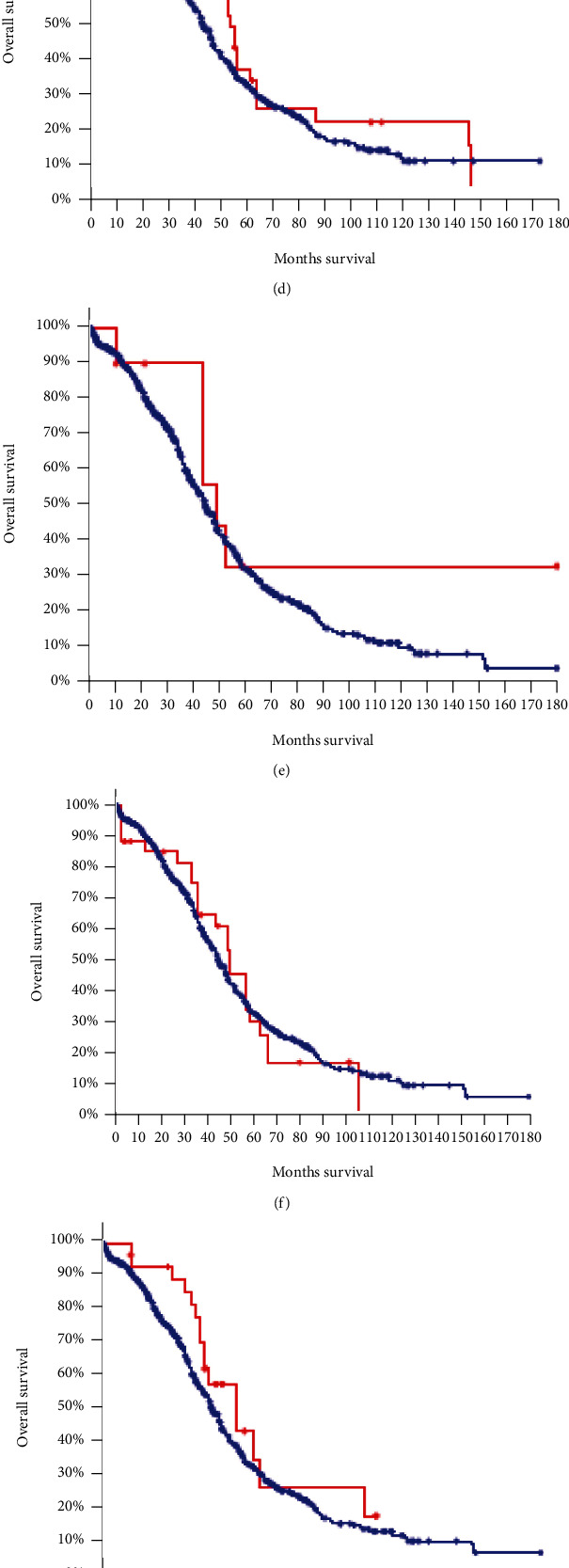
Kaplan-Meier survival curve. The red represents the ovarian cancer group with target gene mutation; blue represents the ovarian cancer group without target gene mutation; *p* value < 0.05 is considered statistically significant.

Through cbioportal analysis of how the genes were correlated with prognosis (Figure 5), we observed improved overall survival rate of ovarian cancer patients with CEP55 variation, with a statistically significant difference (*p* = 0.012). Moreover, patients with CCNE1 variation showed poorer survival prognosis compared to nonvariant tumor patients (*p* = 2.397*e* − 6). However, no significant differences were observed in the survival analysis of other key genes between the two tumor groups.

## 4. Discussion

Ovarian cancer is a common gynecological malignancy. However, the molecular mechanism by which ovarian cancer is associated with pathogenicity has not been fully elucidated. Notably, BRCA is one of the currently identified gene that has a key role in ovarian cancer [21]. The BRCA mutation frequency of ovarian cancer ranges from 3% to 27%; the gene test provides precise guidelines for preventing, diagnosing, and treating ovarian cancer [22]. However, there is a need to identify other novel molecules to jointly screen for most of the remaining cancer cases.

Current researches [10, 23, 24] indicate that besides genes, the dysregulated expression of noncoding RNAs such as miRNA can widely mediate various types of malignant tumors. Therefore, to improve the prediction accuracy, our study identified overlapped DEGs based on integrated miRNA and mRNA expression profiles of ovarian cancer. We constructed the miRNA-gene regulatory network to identify three key miRNAs (miR-195-5p, miR-424-5p, and miR-497-5p) as tumor suppressors based on the principle of complementary binding of miRNAs to the target mRNAs, negatively regulating genes. Moreover, the 10 key genes were predicted and screened by visualizing the overlapped DEGs in the network using the Cytoscape software, whereby TTK, CEP55, and KIT were at the center of the molecular network. By querying the NCBI database, we found that majority of the 10 genes were associated with specific tumorigenesis that mainly involves mitosis and cell proliferation (Table 1). Additionally, the survival curve analysis revealed that CEP55 and CCNE1 should be potential prognostic genes. The biological processes involved in overlapped DEGs were enriched through the R package. It was found that 22.6% (24/106) and 10.4% (11/106) of the DEGs were significantly enriched in biological processes of the reproductive system and DNA transcription activation function, respectively. These results concur with the actual functions and roles of ovarian tissue thereby justifying the reliability of our study. The abnormal expression of these differential miRNAs and genes was likely to mediate the occurrence and development of ovarian cancer. Besides, for the three key miRNAs discovered as tumor suppressors, we conducted an experimental literature search for recently published reports. Notably, Luo et al. [25] identified that the expression of miR-195-5p was significantly reduced in 40 breast cancers through qPCR experiments. Also, he identified CCNE1 was as the direct target of miR-195-5p in a dual-luciferase reporter assay. Elsewhere, Kong et al. [26] found that miR-195-5p played a tumor-suppressive role in endometrial cancer through similar methodologies. Moreover, Liu et al. [27] identified that miR-424-5p directly targeted CCNE1 to inhibit epithelial ovarian cancer through *in vitro* experiments. Notably, we obtained similar findings on the miRNA expression and target prediction. In addition, Liu et al. [28] constructed a lentiviral miR-497-5p system, and through qRT-PCR, she verified that its overexpression enhanced cell apoptosis of ovarian cancer. Similarly, we reported that miR-497-5p as a tumor suppressor molecule.

Conclusively, this study purposed to reveal the key molecules of ovarian cancer by analyzing the integrated miRNA-mRNA expression profiles. Notably, the identified key miRNAs or genes require in-depth experimental verification through *in vitro* studies. Nevertheless, the bioinformatics is a reliable method to predict the expression profiles by narrowing the scope of *in vitro* experiments and saving valuable resources. In the future, we believe that global researchers will be able to instantly reveal key molecules of many complex and diverse tumors using the tumor big data strategy which is dependent on computational biology.

## Figures and Tables

**Table 1:** Function of the top 10 key genes at the hub core of the PPI network.

No	Gene	Full name	Gene functions
1	TTK	TTK protein kinase	Related to cell proliferation, encoding key protein for mitotic checkpoints. Abnormal mitotic spindles are produced when expression is abnormal, resulting in tumor occurring possibly.
2	CEP55	centrosomal protein 55	Playing roles in mitosis and cytokinesis. Related pathways include DNA damage and cytoskeleton signaling.
3	KIT	KIT proto-oncogene	Encoded protein is a type III transmembrane receptor; genetic variation is related to gastrointestinal stromal tumors and mast cells.
4	DTL	denticleless E3 ubiquitin protein	The homologue of E3 ubiquitin protein ligase, which maybe degrade PDCD4 and promote tumor development. It may be a therapeutic target for ovarian epithelial cancer.
5	E2F8	E2F transcription factor 8	A member of family encoding transcription factors, which regulate cell cycle-related gene expression and is involved in the promotion of a variety of tumors
6	SOX9	SRY-box transcription factor 9	Participated in identifying specific sequences. Related to bone deformity, nodular atypical hyperplasia and other diseases.
7	ERCC6L	excision repair 6 like	Members of family encoding protein belong to DNA transport enzymes and are necessary genes for mitotic sister chromatid isolation; involved in cell proliferation.
8	KIF18B	kinesin family member 18B	A member of the kinetin family, which constitutes the main positive end of microtubule depolymerization in mitotic cells, ensuring that the spindle is centered.
9	THY1	Thy-1 cell surface antigen	Encoding cell surface glycoproteins and proteins, involving in adhesion and communication of multiple cell types, which promotes nasopharyngeal carcinoma.
10	CCNE1	cyclin E1	Encoded protein belongs to cell cycle family. Overexpression of gene is observed in many tumors, causing chromosomal instability and may promote tumorigenesis.

Note: The annotations in this table referred to the clear conclusions in NCBI (https://www.ncbi.nlm.nih.gov/gene), and relevant scattered reports weren't included in a single literature.

## Data Availability

All datasets generated for this study are included in the article.
